# Radial Oxygen Loss from the Roots of Mangrove Seedlings Enhances the Removal of Polycyclic Aromatic Hydrocarbons

**DOI:** 10.3390/plants12213711

**Published:** 2023-10-28

**Authors:** Zhimao Mai, Hui Wang, Youshao Wang, Qiqi Chen, Lina Lyu, Xing Wei, Weiwen Zhou, Hao Cheng

**Affiliations:** 1 State Key Laboratory of Tropical Oceanography, CAS Key Laboratory of Tropical Marine Bio-Resources and Ecology, South China Sea Institute of Oceanology, Chinese Academy of Sciences, Guangzhou 510301, Chinawwzhou@scsio.ac.cn (W.Z.); 2 University of Chinese Academy of Sciences, Beijing 100049, China; 3 Daya Bay Marine Biology Research Station, Chinese Academy of Sciences, Shenzhen 518121, China; 4 Coral Reef Research Center of China, Guangxi Laboratory on the Study of Coral Reefs in the South China Sea, School of Marine Sciences, Guangxi University, Nanning 530004, China

**Keywords:** mangrove ecosystem, PAH-ring hydroxylating dioxygenase, microbial community composition, pyrene, root anatomical structure

## Abstract

The presence of polycyclic aromatic hydrocarbons (PAHs) in soil poses a significant global environmental concern, particularly in coastal wetlands. Mangrove ecosystems exhibit enormous potential in environmental purification; however, the underlying mechanisms involved in the degradation of pollutants (e.g., PAHs) remain ambiguous. In the present investigation, a soil pot experiment was conducted with the addition of pyrene to evaluate the effect of radial oxygen loss (ROL) from roots on PAH degradation using three mangrove seedlings (*Rhizophora stylosa*, *Aegiceras corniculatum*, and *Avicennia marina*). The results showed that mangrove plantation can significantly promote the efficiency of pyrene removal. As for the three mangrove species studied, the greatest removal rate (90.75%) was observed in the soils associated with *A. marina*, followed by *A. corniculatum* (83.83%) and *R. stylosa* (77.15%). The higher PAH removal efficiency of *A. marina* can be partially attributed to its distinctive root anatomical structure, characterized by a thin exodermis and high porosity, which facilitates ROL from the roots. The results from qPCR further demonstrate that ROL is beneficial for promoting the abundance of PAH-ring hydroxylating dioxygenase gene, leading to a higher removal efficiency. Additionally, Rhizobiales, Defferrisomatales, and Ardenticatenales may also play important roles in the process of pyrene degradation. In summary, this study provides evidence for elucidating the mechanism of PAH removal from the perspective of ROL, thereby contributing valuable insights for species selection during mangrove restoration and remediation.

## 1. Introduction

Polycyclic aromatic hydrocarbons (PAHs), a kind of organic compounds composed of multiple fused benzene rings, are widely distributed in the environment [1]. The presence of PAHs poses a significant risk to human health due to their carcinogenic, teratogenic, and mutagenic properties [2]. Mangroves serve as a crucial ecological barrier along the coast, playing a pivotal role in the removal of PAHs. The implementation of constructed wetlands has been proposed by numerous scholars as a viable strategy to effectively address pollution [3]. However, the precise mechanism underlying PAH removal by mangroves remains ambiguous. Therefore, it is crucial to identify both intrinsic and extrinsic factors that influence the variability in PAHs removal by mangroves.

Oxidative degradation represents the primary mechanism for eliminating PAHs, and their half-life is prolonged under hypoxic conditions [4]. The initial oxidation of PAHs, especially the oxygenation-induced opening of the benzene ring, represents a crucial step in regulating PAH degradation efficiency [5]. Therefore, soil oxygen concentration is the primary limiting factor for oxidative PAH degradation. Oxygen can be transported from the air to a plant’s roots through its stomata. Excess oxygen is also released into the rhizosphere soil, in addition to supporting aerobic respiration and normal metabolism [6]. The mangrove plants have developed various specialized strategies to thrive in their waterlogged anaerobic habitat through extensive adaptive evolution, including the process of radial oxygen loss (ROL, defined as the transfer of oxygen from root aerenchyma towards the rhizoplane and rhizosphere) [7]. ROL can create a micro-aerobic environment around roots that prevents excessive accumulation of reductive phytotoxins. Our previous study [8] also found that ROL can facilitate N uptake and the growth of mangroves in inter-tidal regions. However, there is a paucity of research on the function of ROL in the epoxidation degradation of PAHs. Previous studies have demonstrated that the capacities of ROL varied significantly among different mangrove species [9]. Therefore, further investigation into whether and how ROL regulates the oxidative degradation of PAHs in various mangrove species would be a valuable pursuit.

The intricate interplay between plant rhizosphere and microorganisms serves as the foundation for pollutant degradation. ROL is a crucial factor influencing the growth and activity of aerobic microorganisms, which may promote PAH biodegradation through oxidative metabolism. The majority of known microorganisms, including bacteria and fungi, possess the ability to secrete PAH-ring hydroxylating dioxygenase (PAH-RHDα) for the biodegradation of PAHs via oxidation pathways [10,11]. Moreover, the gene abundance of the microbial PAH-RHDα gene in soil exhibited a robust positive correlation with its capacity for biodegrading PAHs [12]. Additionally, the degradation efficiency of PAHs by microorganisms is influenced by a range of physicochemical factors, encompassing environmental conditions such as temperature, salinity, and pH [13,14], as well as substrate characteristics including molecular weight and hydrophobicity [15]. Hypothesizing, diverse mangrove species with varying ROL may create distinct rhizosphere environments, which could potentially lead to changes in the biodegradation of PAHs.

Therefore, a soil pot study was carried out to examine the impact of ROL on PAH removal using three mangrove species (*Rhizophora stylosa*, *Aegiceras corniculatum*, and *Avicennia marina*) with varying ROL. The aim of this study is to: (1) assess the efficiency of PAH removal by mangroves with different ROL; (2) elucidate the variations in microbial community composition and PAH-RHDα gene abundance in soils associated with different mangrove planting; (3) identify the correlation between ROL and PAH removal, as well as the related microbial taxa and gene abundance. The findings of this study will contribute to a more comprehensive understanding of the removal of polycyclic aromatic hydrocarbons (PAHs) by mangrove plants from the perspective of ROL.

## 2. Materials and Methods

### 2.1. Experimental Materials and Potted Method

The seeds or propagules of mangrove plants (*R. stylosa*, *A. marina*, and *A. corniculatum*) were collected from Guangzhou, Guangdong province, China one year prior to the commencement of this research. After the process of germination, the young plants were placed in a controlled greenhouse environment with a temperature range of 25 °C and a light intensity of 480 µmol m^−2^ s^−1^. The seedlings of each species were uniform in size, with an average height of approximately 10 cm and a root length ranging from 8 to 12 cm.

The role of ROL in PAH removal was assessed through a pot trial incorporating the addition of pyrene. The potting soil (Table 1) free from PAH contamination was treated with pyrene to achieve a final concentration of 25 ppm. The uniform seedlings of the three aforementioned mangrove plants were transferred into plastic pots (20 cm in diameter and 25 cm in height). Each pot was filled with 4 kg of soil that had been prepared beforehand. The control group was established by preparing another set of pots without plants. The experiment consisted of eight replications for each treatment, and the cultures were kept at 25 °C for 28 days with daily watering of artificial seawater.

### 2.2. ROL Determination and Root Anatomical Analysis

The ROL of three mangrove plants was evaluated utilizing titanium citrate colorimetry. Briefly, the roots of the plant are washed and placed in an anaerobic tank, then immersed in a beaker containing 200 mL of 10% Hoagland’s nutrient solution without oxygen. After fixing the plant, a layer of paraffin wax is applied to the surface to prevent oxygen ingress into the system. The reaction system was then injected with 40 mL of oxygen-free titanium (Ⅲ) citrate solution. The specimen was cultivated under controlled conditions in an artificial climate chamber at 30 °C, with the relative humidity set to 60% and a light intensity of 300 μmol m^−2^ s^−1^ for a duration of 24 h. The control group was established without including plants. After the completion of cultivation, the absorbance of the reaction solution was quantified at 527 nm under anaerobic conditions. The citrate concentration was determined based on the standard curve, while the oxygen influx of herbaceous wetland plant roots was calculated using the method mentioned in the previous study [16]. The ROL from mangrove roots was subsequently determined using the modified Formula (1).

(1) ROL = c (y − z)/4

where the variable c represents the initial volume of titanium citrate (L), while y and z represent the concentrations of titanium citrate (μmol L^−1^) in the control group without plants and the test group, respectively.

As for the measurement of root anatomy, the soils adhering to the roots were meticulously rinsed with distilled water. Subsequently, transverse sections (approximately 5 cm from the tip) were prepared using a freezing microtome (Leica, Benshein, Germany). The area ratio of exodermis, cortex, and stele was then quantified using a microscope (Olympus, Tokyo, Japan), and there were eight replicates for each mangrove species.

### 2.3. Determination of the PAHs in Soil

The pyrene content in the soil was analyzed using GC-MS (Agilent, Santa Clara, CA, USA), following the procedure outlined in the previous study with minor modifications [17]. Briefly, the soil was air-dried naturally, and 1.0 g of the soil samples were transferred into a colorimetric tube after passing through an 80-mesh screen. Subsequently, a mixture of dichloromethane and n-hexane (*v*:*v* = 1:1) along with 0.5 g of copper powder was added to the tube. After sealing, ultrasonic extraction was conducted for a duration of 20 min, followed by collection of the supernatant after allowing it to stand for 30 min. The supernatant was purified using a chromatographic column packed with diatomite, neutral alumina, and anhydrous sodium sulfate. The eluent used was a mixture of dichloromethane and n-hexane (*v*:*v* = 3:7). The purified sample was concentrated and dried using rotary evaporation, followed by GC-MS analysis of the pyrene content to calculate the rate of pyrene removal from the soil.

### 2.4. Microbiological Analysis

#### 2.4.1. Analysis of Quantitative Real-Time PCR

A soil sample weighing 1 g was collected, and the total DNA in the soil was extracted using a soil DNA extraction kit (Omega, Bio-Tek, GA, USA) according to the protocol provided by the manufacturer. Quantitative PCR (qPCR) was performed to estimate the abundance of 16S rRNA genes and PAH-RHDα during the experimental period. The 16S rRNA and PAH-RHDα GP genes were amplified using 338F/518R and 641F/933R primers, respectively [18]. The amplification conditions and reaction systems were mentioned in the previous study [18]. The standard curve was generated by performing tenfold serial dilutions of a known quantity of the plasmid DNA. Each DNA sample was diluted to reduce the impact of inhibitors on threshold cycle suppression for that specific sample type (usually at a ratio of 1:10). The qPCR efficiencies (ranging from 87.9% to 112.5%) were assessed in order to evaluate the presence of any inhibitory effects. The R^2^ values for all calibration curves exceeded 0.99, and the qPCR analysis was conducted utilizing the iCycler (version 3.1.0) software.

#### 2.4.2. 16S rRNA Gene Illumina Sequencing

The V4-V5 region of 16S rRNA genes was amplified using primer pairs 338F/806R, with soil DNA used as the template and employing the protocol mentioned in the previous literature [19]. The products were purified utilizing a purification kit (Omega, D2500-01, Bio-Tek, GA, USA). The Illumina HiSeq platform (Illumina, San Diego, CA, USA) was utilized for high-throughput sequencing by Shanghai Meiji Biomedical Technology Co., Ltd., (Shanghai, China).

The raw sequences were processed using Trimmomatic, and sequence assembly was performed using the FLASH software [20]. Subsequently, the raw data underwent analysis employing the QIIME (version 1.9.1) software and were compared against a reference sequence database [21]. The valid tags were obtained using the UCHIME algorithm [22]. The sequence analyses were conducted using uparesoft (usparse v7.0.1001), and the Silva reference database was employed to annotate each representative sequence with categorical information, ensuring a confidence threshold of ≥0.5 [23].

### 2.5. Statistical and Correlation Analysis

The normality of soil performance data was evaluated using the Kolmogorov–Smirnov test in SPSS version 22.0. A one-way analysis of variance (Tukey-HSD test) was conducted with a significance level of *p* < 0.05 to assess differences in various parameters among soil samples, and multiple comparisons were performed using the least significant difference method. The data obtained were visualized using Origin Pro 2021 and GraphPad Prism 9.0.

The raw reads of 16S rRNA gene sequence were submitted to the NCBI (PRJNA1018813). The bioinformatic and statistical analysis of bacterial taxa was conducted utilizing the accessible packages on Majorbio Cloud Platform (cloud.majorbio.com, accessed on 15 July 2023). The microbial diversity, species richness, and evenness were assessed through the characterization of alpha diversity indices such as the Shannon index, Chao, and Shannoneven index. Additionally, a Venn diagram was utilized to identify unique and common operational taxonomic units (OTUs). The variations in the composition of bacterial communities among multigroup and the comparison between each pair were examined using the Kruskal–Wallis H test. The dominant microbial taxa and their relationship with various parameters were visualized using heatmap analyses.

## 3. Results

### 3.1. The PAH Removal Rate and ROL Values

**Figure 1:**
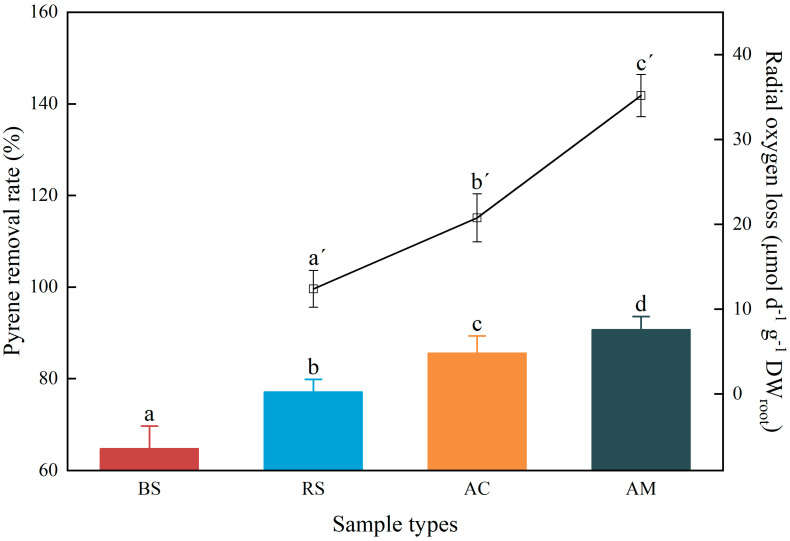
The PAH removal rate and ROL values. The histogram indicates the pyrene removal rate across different sample types, while line graph represents the ROL values from three mangrove species. The mean ± SD is presented, and distinguishing letters indicate significant differences among groups at *p* < 0.05.

After a treatment period of 28 days, the removal rate of pyrene and ROL values in the soil are presented in Figure 1. The removal rate of pyrene was lowest in bare soil without mangrove plants, but significantly increased (*p* < 0.05) to a range between 77.15 and 90.75% in vegetated soil. Notably, *A. marina* exhibited the highest removal rate of pyrene in soil. Consistent with the removal rate of pyrene, *A. marina* also exhibited the highest ROL rate (35.18 µmol d^−1^ DW_root_), followed by *A. corniculatum* (20.77 µmol d^−1^ DW_root_) and *R. stylosa* (12.39 µmol d^−1^ DW_root_).

### 3.2. The Comparative Analysis of Root Anatomy in Three Mangrove Seedling Species

**Figure 2:**
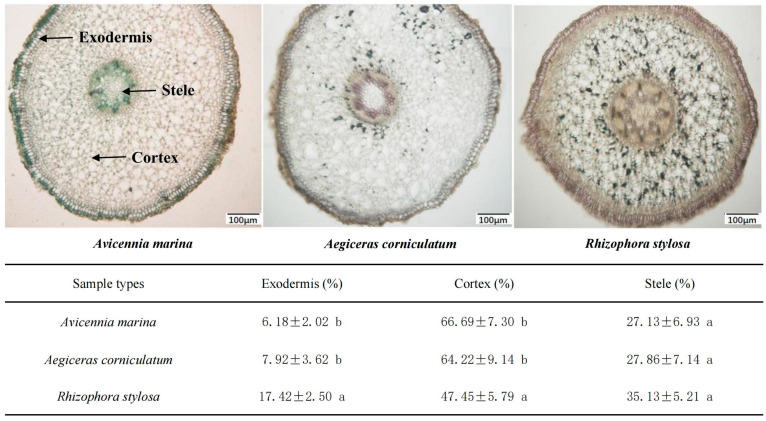
The cross-sectional micrographs of the root base and their anatomical structural characteristics. The mean ± SD is presented, and distinguishing letters represent significant differences among groups at *p* < 0.05.

The root anatomy of three mangrove seedling species is illustrated in Figure 2. The mangrove seedlings displayed prominent aerenchymaunae in the cortex and a compact stele. The *A. marina* displayed the highest proportion of cortex, while *R. stylosa* exhibited the lowest. The proportion of exodermis, however, exhibited an inverse trend, with the lowest observed in *A. marina* and the highest in *R. stylosa*.

### 3.3. The Abundance of PAH-RHDα and 16S rRNA Genes

**Figure 3:**
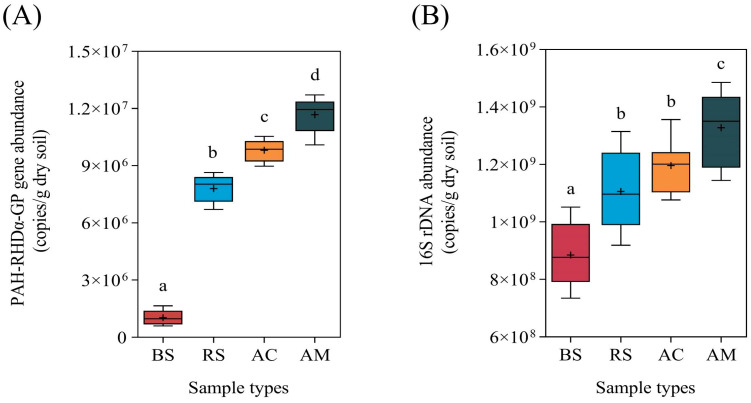
The abundance of PAH-RHDα and 16S rRNA genes. (**A**) The abundance of PAH-RHDα genes. (**B**) The abundance of 16S rRNA gene. The presence of distinct letters indicates noteworthy variances between groups with a significance level *p* < 0.05. Abbreviations: bare soil (BS), *Rhizophora stylosa* (RS), *Aegiceras corniculatum* (AC), *Avicennia marina* (AM).

For the various soil samples, PAH-RHDα GP genes copy number ranged from 1.03 × 10^6^ to 1.17 × 10^7^ copies/dry soil (Figure 3A). The abundance of PAH-RHDα GP genes was significantly elevated (*p* < 0.05) in mangrove-planted soil, with *A. marina* exhibiting the highest abundance among all groups. As shown in Figure 3B, the bacterial abundance was also observed to significantly increase (*p* < 0.05) in mangrove-planted soil. Among the different species, *A. marina* exhibited the highest bacterial abundance, which was 1.5 times greater than that of unvegetated soil.

### 3.4. Correlation between ROL, PAHs Removal, and the Abundance of PAH-RHDα Gene

**Figure 4:**
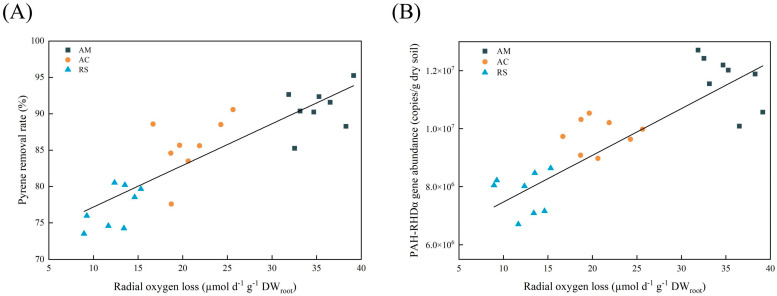
The correlation between radial oxygen loss, pyrene removal rate, and the abundance of PAH-RHDα gene. (**A**) The relationship between radial oxygen loss and pyrene removal rate (y = 0.5726x + 71.452, R^2^ = 0.7559, *p* < 0.01). (**B**) The relationship between radial oxygen loss and the abundance of PAH-RHDα gene (y = 1.50 × 10^5^x + 6.33 × 10^6^, R^2^ = 0.7144, *p* < 0.01). Abbreviations: bare soil (BS), *Rhizophora stylosa* (RS), *Aegiceras corniculatum* (AC), *Avicennia marina* (AM).

**Figure 5:**
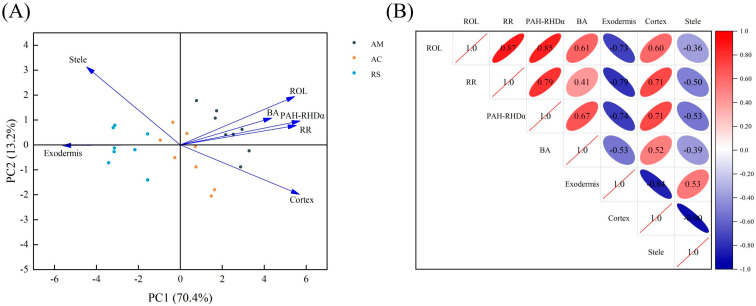
Principal component analysis and Pearson’s correlation analysis of various parameters from different sample types. (**A**) Principal component analysis of various parameters. (**B**) Pearson’s correlation analysis of various parameters. The solid circle scatters represent different sample types, and maroon, blue and orange, and dark blue circle represent the samples from bare soil (BS), *Rhizophora stylosa* (RS), *Aegiceras corniculatum* (AC), and *Avicennia marina* (AM), respectively. Abbreviations: radial oxygen loss (ROL), PAH removal rate (RR), bacterial abundance (BA).

The removal rate of PAHs from soil was observed to be significantly and positively correlated with the ROL from mangrove roots (Figure 4A). Consistently, there were also significant positive correlations between the abundance of PAH-RHDα GP genes and ROL (Figure 4B). The ROL and PAH removal rate were also found to be positively correlated with bacterial abundance and cortex, but negatively correlated with exodermis (Figure 5).

### 3.5. Diversity and Abundance of Microbial Communities

**Figure 6:**
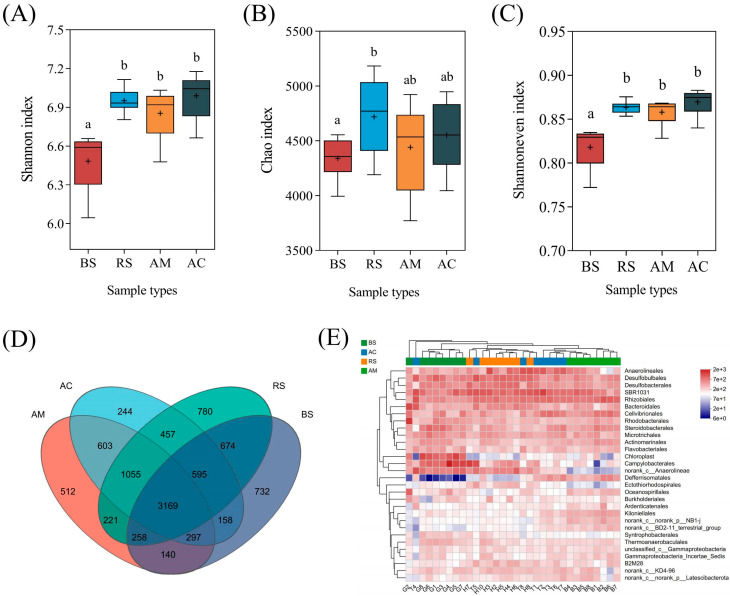
The bacterial community composition and diversity. (**A**) Shannon index. (**B**) Chao index. (**C**) Shannoneven index. (**D**) Venn diagram analysis of OTU numbers among the four groups. (**E**) Heatmap analysis of microbial composition at order level in each sample. The mean ± SD is presented, and different letters represent significant differences among groups at *p* < 0.05. Abbreviations: bare soil (BS), *Rhizophora stylosa* (RS), *Aegiceras corniculatum* (AC), *Avicennia marina* (AM).

After undergoing quality filtering and the removal of chimeric sequences, a total of 648,480 high-quality reads were obtained, resulting in the identification of 105,039 operational taxonomic units (OTUs) with a sequence similarity threshold of 97%. The soil planted with mangroves exhibited a significant enhancement (*p* < 0.05) in microbial diversity and community uniformity compared to the bare soil, while no significant increase in species richness was observed (Figure 6A–C). The Venn diagram analysis (Figure 6D) revealed a total of 3169 common OTUs among the four groups. The bacterial relative abundance at order level is shown in Figure 6E; Rhizobiales (2.73–4.78%), Campylobacterales (0.85–6.41%), Anaerolineales (2.15–4.54%), Desulfobacterales (2.46–4.25%), and Cellvibrionales (1.97–2.62%) are the predominant bacterial taxa in soil samples. 

### 3.6. The Variation in Microbial Compositions and Potential Bacterial Taxa Contributes to PAHs Removal

**Figure 7:**
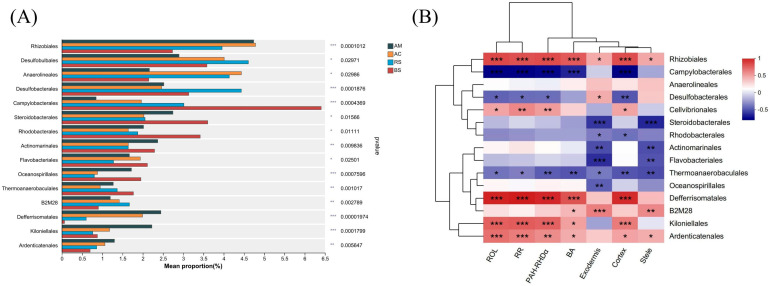
The potential bacterial taxa that could potentially contribute to the alterations in PAH-RHDα and PAH degradation. (**A**) The variation in bacterial composition among multiple groups. (**B**) The correlation analysis of the dominant bacterial taxa (at the order level) with PAH-RHDα and PAH removal. Abbreviations: bare soil (BS), *Rhizophora stylosa* (RS), *Aegiceras corniculatum* (AC), *Avicennia marina* (AM), radial oxygen loss (ROL), PAH removal rate (RR), bacterial abundance (BA).

Figure 7A illustrates the variations in bacterial composition at the order level. The relative abundance of dominant bacterial taxa such as Rhizobiales, Anaerolineales, Desulfobacterales, and Cellvibrionales varied significantly among multigroup. Compared to soil without mangrove plants, the relative abundance of dominant bacterial taxa such as Rhizobiales, Defferrisomatales, and Ardenticatenales was higher in soil planted with mangroves. The greatest prevalence of Defferrisomatale and Ardenticatenales were found in soil planted with *A. marina*.

The bacterial taxa with potential contribution to PAHs removal are illustrated in Figure 7B. The dominant microbial taxa, including Rhizobiales, Defferrisomatales, Kiloniellales, and Ardenticatenales, exhibited a positive correlation with the removal rate of PAHs. Additionally, these bacterial taxa were also positively correlated with PAH-RHDα and ROL, but negatively correlated with exodermis.

## 4. Discussion

### 4.1. Variations in PAH Removal in Soil Associated with Different Mangrove Plantations

The results showed that mangrove plantation significantly enhances the removal rate of pyrene in soils, resulting in a significant increase from 64.81 to 90.75%, compared to bare soil without mangroves (Figure 1). The present finding is consistent with prior research that suggests incorporating vegetation can significantly enhance the removal of PAHs in soil [24]. For instance, the cultivation of ryegrass significantly enhanced the removal of pyrene from soil after an 8 week period [25]. A study utilizing *Bidens maximowicziana* to evaluate the potential of pyrene pollution remediation demonstrated that planted soil had a significantly higher pyrene removal rate compared to non-planted soil, with an average removal rate of 79% after 50 days of growth, representing a 28% increase over non-planted soil [26]. In the current study, the greatest pyrene removal rate was observed in the soils associated with *A. marina*, whereas *R. stylosa* exhibited the lowest removal efficiency (Figure 1). The previous study also demonstrated that *Avicenn marina* exhibits a higher capacity for the absorption and accumulation of PAHs compared to other mangrove species, attributed to its greater root length and specific root length [27].

Notably, the removal efficiency of pyrene by mangrove plants was found to be positively correlated with their ROL capacity (Figure 4A and Figure 5). Previous researchers also proposed a similar phenomenon, that ROL from the roots of wetland plants appeared to facilitate the removal of nutrients (such as TN and NH_4_-N) [28] and Cd [29] from waste waters. All of these results indicate that the capacity of ROL can serve as an effective biological indicator for plant screening during phytoremediation. In this study, mangrove plants, especially *A. marina*, which demonstrate powerful ROL hold promising potential for remediating PAH pollution. The previous studies have also claimed that *A. marina* exhibits a higher ROL than other true mangrove species [30]. Nevertheless, both ROL and pyrene removal were also found to be highly related to root features (Figure 2). In this study, the remarkable ROL of *A. marina* can be largely attributed to its unique root anatomical structure, characterized by extensive root porosity that facilitates efficient diffusion of oxygen within the roots. Additionally, a thinner exodermis can decrease the resistance to transverse diffusion of oxygen across the outer cell layers, resulting in an increased rate of oxygen release from the roots of *A. marina*. 

### 4.2. The Importance of ROL in PAH Removal

ROL is considered as an importance adaptive strategy for wetland plants in resisting waterlogging. Frequent tidal inundation can easily lead to soil hypoxia and the accumulation of reductive phytotoxins (e.g., S^2−^, HS^−^, and Fe^2+^). ROL from roots can create an aerobic micro-rhizosphere, which may alleviate phytotoxicity induced by waterlogging. Our previous study [8] further emphasized the significance of ROL in regulating nitrogen translocation and transformation at the interface between roots and soil. It was found that ROL plays a role in mitigating nitrate deficiency by promoting the growth of ammonia-oxidizing bacteria and archaea in the rhizosphere, ultimately facilitating nitrogen uptake by mangroves. Additionally, ROL can promote the formation of iron plaque coating on the root surface, which plays a crucial role in metal detoxification and nutrient uptake [31]. All of these benefits attributed to ROL facilitate the growth and biomass yield of mangroves, potentially leading to increased absorption of PAHs from soils [27].

Moreover, ROL creates a micro-oxidative environment that enhances the abundance of microorganisms involved in PAH degradation. Aerobic biodegradation is the predominant microbial metabolic pathway for PAHs, exhibiting greater efficiency than anaerobic biodegradation [32]. The oxidative ring-opening of the benzene ring, catalyzed by aerobic microorganisms through oxygenase secretion (e.g., PAH-RHDα), represents a crucial and initial step in the biodegradation process of PAHs [33]. The results from Figure 4B clearly showed a positive correlation between ROL and the abundance of PAH-RHDα. Notably, soils associated with *A. marina*, which exhibited the highest ROL among the three mangrove species studied, consistently displayed the greatest prevalence of PAH-RHDα genes (Figure 3A). The high prevalence of PAH-RHDα genes in soil and sediment samples indicates a greater potential for the biodegradation of PAHs [18,34]. Thus, the highest pyrene removal in *A. marina* (Figure 1) may be attributed to the enhancing PAH-RHDα genes facilitated by ROL. Nevertheless, more detailed investigation on the potential correlations between ROL and other genes (e.g., phenol monooxygenase and catechol dioxygenase genes) involved in PAH degradation is also warranted. Additionally, accurate and comprehensive analysis of PAH degradation and oxygen dynamics in the rhizosphere at a smaller micro-level would also be worth exploring.

Additionally, both ROL and PAH removal are closely linked to microbial communities composition. Although the dominant bacterial taxa were similar across all groups, there were significant variations in their relative abundances (Figure 6E and Figure 7A). In the current research, variation analysis of bacterial composition revealed a higher abundance of Rhizobiales, Defferrisomatales, and Ardenticatenales in mangrove-planted soil (Figure 7A). Positive correlations between these microbial taxa and PAH removal (Figure 7B) indicated a potential contribution to the biodegradation of PAHs. Rhizobiales, a plant symbiont commonly found in the mangrove rhizosphere, facilitates plant growth by providing nutrients, plant hormones, and essential metabolite precursors that are beneficial for PAH removal [35,36]. A previous study has revealed that a predominant bacterial group, closely related to Rhizobia, capable of degrading PAHs was detected in the soil treated with the bioreactor [37]. Similarly, it has been reported that the addition of wheat straw biochar can enhance the abundance of Ardenticatenales, thereby facilitating the degradation of organic pollutants such as PAHs in sediment [38]. Other potential bacterial groups, such as Defferrisomatales and Kiloniellales, may also be significant in the degradation of PAHs. However, the mechanism underlying PAH degradation driven by these microbial groups remains poorly understood, necessitating further research to isolate, culture, and investigate the degradation capabilities of these microorganisms.

## 5. Conclusions

The current investigation provides evidence for the potential role of ROL in promoting the degradation of PAHs through oxidative cleavage of the benzene ring. The cultivation of mangrove, especially *A. marina*, significantly promoted the efficiency of pyrene removal. The enhanced PAH removal efficiency can be partly attributed to their unique root anatomical structure (e.g., a thin exodermis together with high porosity) that benefits ROL from roots. An interesting positive linkage between pyrene removal and ROL was also observed. The present data further substantiate the role of ROL in enhancing the gene abundance of PAH-RHDα. Additionally, Rhizobiales, Defferrisomatales, and Ardenticatenales may be also significant in PAH degradation. This study offers valuable insights for guiding species selection during mangrove restoration and management.

## Figures and Tables

**Table 1:** General properties of the potting soil collected from Guangzhou (mean ± SD, *n* = 8).

Parameters	Values
pH	6.9 ± 0.3
Sand (%)	33 ± 2.4
Sitl (%)	43 ± 3.2
Clay (%)	24 ± 1.8
Organic matter (%)	5.7 ± 2.1
